# Draft Genome Sequence of *Mycobacterium bovis* Strain SP38, a Pathogenic Bacterium Isolated from a Bovine in Brazil

**DOI:** 10.1128/genomeA.00511-15

**Published:** 2015-05-21

**Authors:** Ana Marcia Sa Guimaraes, Cristina Kraemer Zimpel, Cássia Yumi Ikuta, Naíla Cannes do Nascimento, Andrea Pires dos Santos, Joanne Belle Messick, Marcos Bryan Heinemann, José Soares Ferreira Neto, Paulo Eduardo Brandão

**Affiliations:** a Department of Preventive Veterinary Medicine and Animal Health, University of São Paulo, São Paulo, Brazil; b Department of Comparative Pathobiology, Purdue University, West Lafayette, Indiana, USA

## Abstract

We report a draft genome sequence of *Mycobacterium bovis* strain SP38, isolated from the lungs of a cow in Brazil. The assembly of reads resulted in 36 contigs in a total of approximately 4.37 Mb. Comparison of *M. bovis* strains sequenced to date will aid in understanding bovine tuberculosis in Brazil.

## GENOME ANNOUNCEMENT

*Mycobacterium bovis* is the causative agent of bovine tuberculosis, an OIE (World Organisation for Animal Health) notifiable disease, responsible for major economic losses in livestock. Bovine tuberculosis is a contagious, infectious disease that can affect several domestic and wild animals besides human beings. In Brazil, the last evaluation of the National Program for the Control and Eradication of Animal Tuberculosis indicated a low prevalence of bovine tuberculosis (~1.3%). The prevalence of *M. bovis* infection in humans, however, is unknown. To better understand the bacterial maintenance in diverse populations and the transmission involving several animal species, it is necessary to comprehend the metabolism and mechanisms of pathogenicity of national *M. bovis* strains by comparing them to other sequenced strains*.* Therefore, the goal of the present study was to sequence and annotate the complete genome of *M. bovis* SP38, a Brazilian strain isolated from an infected bovine.

*M. bovis* was isolated from the lungs of an infected cow from a slaughterhouse in Sao Paulo, Brazil, following standard procedures (1) (IACUC protocol 1628/2009). DNA was extracted according to previous protocols (2, 3) and whole-genome sequencing from a paired-end library (TruSeq DNA sample preparation kit; Illumina, CA, USA) was performed using Illumina HiSeq2500. Reads were assembled using ABySS version 1.2.7 and Projector2. First-pass annotation was obtained using the NCBI pipeline. BLASTclust (4) was used to identify paralogous gene families (PGF) and predicted protein-coding sequences (CDSs) were compared to a set of proteins previously identified in the Brazilian and United Kingdom PPD (purified protein derivative of tuberculin from *M. bovis* strain AN5) (5) using BLAST (6).

A total of 29,651,856 reads were assembled into 36 contigs, resulting in a genome size of ~4.37 Mb with a GC content of 65.6%. First-pass annotation resulted in 3,986 CDSs, 3 rRNAs, and 45 tRNAs. Of these CDSs, 1,059 (26.57%) were annotated as hypothetical proteins and the remaining 2,927 (73.43%) had putative functions. A total of 308 PGFs, with 2 to 124 protein members each, were identified, comprising a total of 1,089 CDSs (27.3%). These numbers are similar to those obtained from other *M. bovis* strains deposited in GenBank. Orthologous CDSs to all 116 PPDs were identified in the *M. bovis* strain SP38 and *M. tuberculosis* strain H37Rv. Low protein-sequence variability was detected in 8 of the SP38 orthologs and in 27 of the H37Rv orthologs (98% to 100% identity, 100% coverage), while moderate variability was observed in only 1 SP38 ortholog (95% identity, 100% coverage) and in 3 H37Rv orthologs (89 to 95% identity, 48 to 98% coverage). Nevertheless, 43 (37%) of these proteins are grouped into 37 PGFs in the SP38 strain, and differential gene expression has been already described for some of these families (7–10). In conclusion, differential gene expression within PGFs should be investigated as a possible source of host-bacterium relationship diversity and tuberculin skin test variation. Further analyses of this genome will provide information about *M. bovis* SP38 biology compared to other *M. bovis* strains.

### Nucleotide sequence accession numbers. 

The *Mycobacterium bovis* SP38 sequence was deposited in GenBank under the accession number JXOQ00000000. The version described in this paper is version JXOQ00000000.1.
